# Supplementary material for: The causative role of amyloidosis in the cardiac complications of Alzheimer's disease: a comprehensive systematic review
Source: J Physiol. 2025 May 5;604(4):1646–81. doi: 10.1113/JP286599 (PMC12909685; doi:10.1113/JP286599)
Supplement: Supplementary file 2 — Supplementary Table – A summary of the publications used in our systematic review. [file TJP-604-1646-s001.docx]

**The causative role of amyloidosis in cardiac complications of Alzheimer’s disease: a comprehensive systematic review**

Samuel Parker, Andrew James, Svetlana Mastitskaya

***Supplementary Table –*** ***A summary of the publications used in our systematic review.***

*AD, Alzheimer’s disease; APOE, apolipoprotein E; APP, amyloid precursor protein; ATP, adenosine triphosphate; ATTR, transthyretin amyloidosis; BACE1, β-secretase; CPB, cardiopulmonary bypass; CSF, cerebrospinal fluid; FRS, Framingham Risk Score; GFAP, glial fibrillary acidic protein; HPA, hypothalamic-pituitary-adrenal axis; HRV, heart rate variability; MMSE, mini mental state examination; NFTs, neurofibrillary tangles; NLRP3, PYD domains-containing protein 3; POCD, post-operative cognitive dysfunction; PS1, presenilin 1; PS2, presenilin 2; p-tau, hyperphosphorylated tau; RSA, respiratory sinus arrhythmia; t-tau, total tau; ΔΨm, mitochondrial membrane potential.*

| Author | Study type | Important findings |
| --- | --- | --- |
| Cardiogenic dementia | | |
| Cardiac arrest increases vulnerability to Alzheimer’s disease | | |
| (Wiśniewski & Maślińska, 1996) | Human observational study of 12 patients | Aβ deposition, neuronal loss, and neuroinflammation were found in brain specimens of those who suffered cardiac arrest |
| (Zetterberg *et al.*, 2011) | Human observational study of 25 patients | Patients who suffered cardiac arrest showed a 7-fold increase in serum levels of Aβ42. Longer duration of Aβ42 elevation correlated to poorer outcomes. |
| (Ashton *et al.*, 2023) | Human observational study in 717 patients | In patients with poor neurological function after cardiac arrest, p-tau in blood was elevated at all timepoints, but reduced over time. Aβ42 and Aβ40 showed delayed increases. |
| (Kocki *et al.*, 2015) | Murine study *in vivo* | Hippocampal PS1 expression increased by over 2-fold, PS2 by over 3-fold, BACE1 increased nearly 4-fold, and APP expression decreased by 0.5-fold, two days after cardiac arrest induction. |
| Cardiopulmonary bypass may induce neuronal injury and cerebral amyloidosis | | |
| (Wang *et al.*, 2022a) | Human observational study in 82 patients | Patients underwent aortic surgery with CPB. In the POCD group, CSF Aβ42 decreased compared to the baseline and the non-POCD group. P-tau and t-tau increased similarly in both groups. Serum Aβ42, p-tau, and t-tau were unchanged. |
| (Požgain *et al.*, 2022) | Human observational study in 54 patients | Serum Aβ42 significantly increased after CPB in patients with coronary artery disease, more so than surgery without CPB. Aβ42 correlated with POCD and poorer memory recall. |
| (Klinger *et al.*, 2018) | Human observational study in 40 patients | POCD present in 35%, 57% and 44% of patients at 6 weeks, 1 year and 3 years, respectively, in patients undergoing CPB. Cerebral Aβ in brain PET imaging was similar in patients with and without POCD at 6 weeks and 1-year post-surgery. |
| (Hu *et al.*, 2016) | Human observational study in 42 patients, <3 years old | Serum Aβ40 and Aβ42 levels were lower up to 6 hours post-surgery with CPB in infants. Pre-operative Aβ42 correlated to intensive care length of stay. |
| (Reinsfelt *et al.*, 2013) | Human observational study in 10 patients | CSF Aβ42 increased by 43% and sAPP-β declined by 31% after CPB, but sAPP-α was unaffected. |
| (Evered *et al.*, 2009) | Human observational study in 332 patients | POCD present in 13% of patients both at 3- and 12-months post-cardiac surgery with CPB. Patients with POCD at 3 months had lower plasma Aβ40 and Aβ42 levels than the non-POCD group, with no difference at 12 months. |
| (Sparks *et al.*, 2000) | Human observational study in 342 patients, and *in vivo* porcine study | Mitral valve disease associated with increased cerebral Aβ burden in patients undergoing CPB. Pigs undergoing CPB showed increased Aβ deposition compared to non-CPB pigs, but Aβ burden was similar regardless of the quality of cardioprotection. |
| (Palotás *et al.*, 2010) | Human observational study in 30 patients | Significant increases in all AD biomarkers in CSF were observed 1-week post-surgery with CPB. At 6 months, tau continued to increase whereas the Aβ peptide levels declined. S100β levels increased post-surgery. |
| (Alifier *et al.*, 2020) | Human observational study in 51 cardiac surgery and 16 MI patients | Blood tau and neurofilament light were higher in patients undergoing cardiac surgery compared to otolaryngeal surgery, Aβ40 and Aβ42 remained unchanged. The presence of myocardial infarction did not associate with tau or neurofilament light release. |
| The effect of heart failure on Alzheimer’s pathology | | |
| (Baranowski *et al.*, 2021) | Porcine study *in vivo* | In pigs with induced heart failure, cerebral blood flow and hippocampal Aβ42 were diminished, whereas APP, BACE1, C99, and Aβ40 were increased compared to controls. The animals’ cognitive abilities were not investigated. |
| (Hong *et al.*, 2013) | Murine study *in vivo* | Female mice with heart failure and left ventricular enlargement showed upregulation of BACE1, APP, and more cognitive dysfunction compared to males. Heart failure had no impact on Aβ or tau deposition in either sex. |
| (Santos *et al.*, 2016) | Human observational study in 63 patients | A higher rate pressure product at rest correlated with greater Aβ amyloidosis. In subjects with evidence of Aβ deposition, the rate pressure product increased by 11.2 mmHg*bpm and was associated with poorer cognition. |
| (Johansen *et al.*, 2019) | Human observational study in 306 patients | For every 1cm increase in left ventricle end-diastolic volume, the odds of Aβ deposition increased (odds ratio 1.76). This association was only present in patients <75 years old. Other echocardiographic parameters were not associated with Aβ. |
| (Tin *et al.*, 2023) | Human observational study in 4037 patients | Levels of NPPB, IBSP and THBS2 were associated with levels of circulating Aβ peptides and incident dementia in later life. NPPB is a known biomarker of heart failure, IBSP is upregulated in aortic valve stenosis and carotid atherosclerosis, and THBS2 is upregulated in aortic valve stenosis too. |
| (Lindbohm *et al.*, 2022) | Human observational study in 13669 patients | N-terminal pro-BNP concentrations were associated with increased odds of dementia in the Whitehall II study (odds ratio 1.52) and the ‘Atherosclerosis risk in communities’ study (odds ratio 1.08). |
| (Hu *et al.*, 2024) | Human observational study in 1244 patients | Left atrial size, indexed for body surface area, positively correlated to CSF p-tau, but not to CSF levels of Aβ peptides. Similarly, a greater left ventricle size correlated to higher CSF p-tau levels, but also lower CSF Aβ42/Aβ40. These echocardiographic parameters are also associated with reduced cognitive function on Montreal cognitive assessment. |
| (Zheng *et al.*, 2021) | Human observational study in 423 patients | In patients without cognitive impairment, lower left ventricular ejection fraction correlated to higher CSF t-tau, but not Aβ42. Lower left ventricular ejection fraction associated with higher CSF p-tau in individuals <65 years old. |
| (Ebenau *et al.*, 2023) | Human observational study in 187 patients | Patients with a lower cerebral blood flow exhibited more cerebral Aβ amyloidosis. Conversely, patients with a higher amyloid burden at baseline experienced greater declines in cerebral perfusion. |
| (Trieu *et al.*, 2024) | Human observational study in 540 patients | Neurofilament light and GFAP were higher in the patients with vascular pathology than the healthy controls, but Aβ42/Aβ40 and p-tau levels were similar between groups. In patients with heart failure, p-tau and GFAP levels were not significantly associated with longitudinal cognitive decline. In patients with carotid artery disease, only p-tau was associated with poorer executive functioning over time; none of the other biomarkers were associated with cognitive decline. |
| (Nural-Guvener *et al.*, 2013) | Murine study *in vivo* | MI was induced through ligation of the left anterior descending coronary artery, and this led to heart failure in the mice alongside a rise in left ventricular end-diastolic pressure and left ventricular remodelling. Heart failure led to microglial activation in the cortex, but not the hippocampus. Whilst myocardial infarction did not immediately upregulate BACE1, the development of heart failure resulted in BACE1 upregulation in the cortex and hippocampus. |
| (Kresge *et al.*, 2020) | Human observational study in 152 patients | Higher left ventricular ejection fraction correlated with lower CSF Aβ42. Excluding patients with cardiovascular disease and atrial fibrillation made this finding statistically insignificant. Lower left ventricular ejection fraction associated with greater CSF t-tau and p-tau in patients with normal cognition. |
| (Dewan *et al.*, 2024) | Human observational study in 2895 patients | 10% of patients with heart failure with preserved ejection fraction had MMSE scores of <24. The mean left ventricular ejection fraction was 57%. Pharmacotherapy with valsartan or a combination of sacubitril and valsartan therapy did not influence cognition. |
| (Kaufman *et al.*, 2021) | Human observational study in 54 patients | A reduction in the brain’s perfusion, shown by a lower cerebrovascular conductance when the patient was at rest, predicted a higher degree of Aβ deposition in patients carrying the APOE-ε4 allele. This relationship was not present in patients without this allele. |
| (Miners *et al.*, 2018) | Human observational study in 86 autopsy specimens | Brain specimens from patients with AD showed reduced levels of platelet-derived growth factor receptor-β, a pericyte marker, compared to control samples (p=0.0002). Elevated levels of fibrinogen were noted in AD brains (p=0.0026), indicating blood-brain barrier breakdown. Levels of platelet-derived growth factor receptor-β negatively correlated with levels of vascular endothelial growth factor, and positively correlated with MAG:PLP1, with a decline in this ratio indicating reduced oligodendrocyte oxygenation and thus cerebral hypoperfusion. Loss of pericytes and breakdown of the blood-brain barrier associated with Aβ plaque amyloidosis in the precuneus. |
| Orthostatic hypotension increases susceptibility to dementia | | |
| (Zhang *et al.*, 2021) | Human observational study in 274 patients | Patients with orthostatic hypotension had higher Aβ42, t-tau and p-tau levels compared to controls without orthostatic hypotension, but only p-tau showed statistical significance. |
| (Ruiz Barrio *et al.*, 2023) | Human observational study in 269 patients | Early-onset orthostatic hypotension in patients with Parkinson’s Disease increased risk of dementia by 14% annually but did not associate with Aβ or tau amyloidosis. |
| Atherosclerosis and its risk factors precede Alzheimer’s disease neuropathology | | |
| (Vargas-Soria *et al.*, 2022) | Murine study *in vivo* | High-fat diet feeding caused more severe cerebral amyloid angiopathy in AD mice with type 2 diabetes compared to mice with prediabetes. This angiopathy affected larger vessels in diabetes than prediabetes. The oxidative stress and matrix metalloproteinase activity induced by cerebral amyloid angiopathy is greater in diabetic mice. |
| (Vemuri *et al.*, 2017) | Human observational study in 430 patients | Patients with arrhythmias, coronary artery disease, heart failure, or diabetes had significantly thinner cerebral cortices, but no cardiovascular disease-influenced cerebral Aβ or tau amyloidosis. |
| (de Silva *et al.*, 2022) | Human observational study in 730 patients | The AD-polygenic risk score and coronary artery disease-polygenic risk score associated with a decline in brain volume in patients with mild cognitive impairment. These associations were not present in patients with normal cognition or AD. |
| (Vescio & Pattini, 2024) | A search in a Genome-wide association study catalogue | Genetic variants of genes associated with CAD interacted with genes involved in lipoprotein clearance, arterial morphology, and inflammation. Furthermore, highly connected nodes in the network analysis included APP and huntingtin, associated with AD and Huntington’s disease. |
| (Seppälä *et al.*, 2010) | Human observational study in 806 patients | Coronary heart disease in the participants was associated with a greater plasma Aβ40 level (p=0.035). However, patients who experienced cognitive decline showed reduced plasma Aβ42 and Aβ42/Aβ40 ratios. |
| (Sparks, 1997) | Human observational study in 138 autopsies | Patients with critical coronary artery disease who carried the ApoE-ε4 allele, and did not have AD, had significantly more Aβ senile plaques in the frontal pole and parahippocampal gyrus, but no statistically significant difference in the number of NFTs. |
| (Liu *et al.*, 2019) | Human observational study in 83 patients | A 1% increase in flow-mediated dilation, where a lower value indicates greater cardiovascular disease risk, correlated to a decrease in Aβ deposition by units of standardised uptake value ratio 0.03. |
| (Twait *et al.*, 2024) | Human observational study in 594 patients | Patients with cerebrovascular disease, CAD, peripheral artery disease or abdominal aortic aneurysm were recruited. Greater levels of neurofilament light are associated with greater odds of having a cerebral infarct (odds ratio 1.42, p=0.039) and lower brain volume. Higher GFP levels are associated with increased odds of cortical infarction (odds ratio 1.45, p=0.010). Only Aβ40 levels are associated with a reduction in hippocampal volume. |
| (Li *et al.*, 2024) | Human observational study in 1464 patients | Non-cognitively impaired patients with more cardiometabolic diseases such as hypertension, diabetes, or stroke exhibited higher levels of CSF p-tau and t-tau (p=0.047). |
| (Baradaran *et al.*, 2022) | Human observational study in 47 patients | Patients without dementia who had higher carotid intima-media thickness on ultrasound scanning exhibited higher Aβ burden, particularly in the precuneus, frontal, anterior cingulate, orbitofrontal, temporal and parietal regions. However, change in intima-media thickness did not associate with Aβ burden, and carotid stenosis did not correlate to Aβ amyloidosis. |
| (Kučikienė *et al.*, 2022) | Human observational study in 838 patients | Patients with abnormal CSF AD biomarkers and magnetic resonance imaging evidence of AD were more likely to have CHD and carotid artery stenosis. Also, carotid artery stenosis associated with greater levels of Aβ42 and t-tau. |
| (Wolters *et al.*, 2022) | Human observational study in 3879 patients | Aβ40 independently associated with coronary artery and carotid artery calcification. Higher levels of Aβ40 related to an increased risk of atherosclerotic cardiovascular disease (hazard ratio 1.10), but after adjusting for baseline risk factors this association became non-significant. |
| (Frentz *et al.*, 2024) | Human observational study in 2,229 patients | Both arterial calcification and plasma Aβ42 levels are independently associated with poorer cognition. Levels of Aβ42 and calcification interacted, where amyloid affected cognition to a greater degree in individuals with more arterial calcification. After adjusting for factors such as smoking, blood pressure, diabetes mellitus, and serum cholesterol, this association was diminished. |
| (Shabir *et al.*, 2022) | Murine study *in vivo* | Atherosclerosis was induced in mice to create a PCSK9-ATH model. This model showed reduced levels of oxyhaemoglobin compared to wild-type and mixed model mice (mice where both atherosclerosis and AD were induced). Cortical spreading depression was worse in mice with atherosclerosis and AD, a phenomenon which should not occur in healthy brain tissue. Mixed model mice demonstrated a greater burden of Aβ plaques and greater levels of neuroinflammation. |
| (Zhang & Luo, 2020) | Murine study *in vivo* | Myocardial infarction was induced in AD-prone APPswe/PS1de9 mice and wild-type C57/BL6 mice. APPswe/PS1de9 mice exhibited poorer behaviour in the social recognition test than C57/BL6, after myocardial infarction. Both strains demonstrated poorer habituation and dishabituation responses to stimuli compared to sham group mice. Myocardial infarction increased cerebral reactive oxygen species production. Hippocampal Aβ and p-tau amyloidosis, and microglia-mediated neuroinflammation. |
| (Cheng *et al.*, 2021) | Murine study *in vivo* | Myocardial infarction was induced in mice using left anterior descending artery ligation. Ligation or sham procedures were performed in wild type, APPswe/PS1de9 mice and APPswe/PS1de9 mice who were knocked out for PYD domains-containing protein 3 (NLRP3). APPswe/PS1de9 mice showed less impairment in spatial reference memory during the Morris water-maze test and less impairment in the locomotor test for motor activity than APPswe/PS1de9/NLRP3 knockout mice after ligation. Cerebral Aβ levels were lower in APPswe/PS1de9/NLRP3 knockout mice than in APPswe/PS1de9 mice after coronary artery ligation. |
| (Robinson *et al.*, 2022) | Human observational study in 1485 autopsies | Vascular risk factors include but are not limited to, atherosclerosis, diabetes, hypertension and arrhythmias associated with an increased number of cerebral infarcts and cerebral arteriolosclerosis. These factors were not associated with cerebral amyloid angiopathy. Patients with evidence of AD pathology were more likely to have multiple vascular risk factors than non-AD controls. |
| (Kosunen *et al.*, 1995) | Human observational study in 38 autopsies | Specimens from patients with AD showed that those carrying the ApoE-ε4 allele demonstrated more severe coronary atherosclerosis than non-carriers with AD. However, in the specimens from patients with confirmed AD, those with more severe coronary atherosclerosis showed similar numbers of Aβ plaques to patients with milder or no coronary atherosclerosis. Similar results were obtained concerning cerebral artery atherosclerosis. |
| (Alafuzoff & Libard, 2020) | Human observational study in 119 patients | Aβ amyloidosis, but not tauopathy, was more common in brain specimens from cognitively impaired patients. The authors could not confirm that cardiovascular disease predisposes to AD amyloidosis. |
| (Moore *et al.*, 2022) | Human observational study in 142 patients | The left ventricular mass index did not correlate to CSF Aβ or tau. In individuals with mild cognitive impairment and APOE-ε4 carriers, left ventricular mass index positively correlated to neurofilament light. |
| (Hendrickx *et al.*, 2022) | Murine study *in vivo* | AngII administration induced hypertension and ventricular hypertrophy, did not worsen Morris Water Maze performance in hAPP23+/- or hAPPswe/PS1dE9 mice compared to control mice. Although AngII reduced visuospatial performance and memory. Histological analysis revealed that hypertension did not increase cerebral Aβ burden. |
| (Smith *et al.*, 2018) | Human observational in 67 patients | Participants with subjective cognitive issues and mild cognitive impairment had similar positron emission tomography evidence of Aβ and similar white matter hyperintensity volumes. In both groups, higher cerebral Aβ burden associated with poorer episodic memory. Although, the presence of hypertension did not affect Aβ deposition shown on positron emission tomography. |
| (Lu *et al.*, 2024) | Human observational study in 1525 patients | Patients with hypertension exhibited a higher rate of increase in p-tau and neurofilament light from midlife to later life than normotensive individuals. Patients with diabetes showed slower increases in Aβ42:Aβ40 and greater neurofilament light than non-diabetic patients. Coronary heart disease did not associate with these two biomarkers. Lower Aβ42:Aβ40, higher p-tau, and higher neurofilament light associated with a greater incidence of dementia in later life. |
| (Streit & Sparks, 1997) | Human observational study in 25 autopsies and rabbit study | In non-dementia patients with >75% stenosis of the coronary arteries, cerebral microglia were more activated than control patients without heart disease. Hypercholesterolaemia in rabbits also increased microglia activation and leukocyte infiltration. Both pathologies associated with greater deposition of Aβ protein. |
| Hypertension increases Alzheimer’s disease biomarkers, neurodegeneration and cognitive decline | | |
| (Csiszar *et al.*, 2013) | Murine study *in vivo* | AngII administration induced hypertension in C57/BL6 mice. Old hypertensive mice demonstrated poorer performance in the Y-maze and novel object recognition tests than normotensive mice. Hypertension did not alter the expression of APP, PS1, PS2, or γ-secretase and reduced the expression of BACE1 in the hippocampus. Hypertension in older mice downregulated acetylcholine and glutamate receptors. Older mice with hypertension exhibited reduced expression of cyclin-dependent kinase 5 and protein kinase N1. Finally, hypertension increased hippocampal caspase 3 and Apcs expression. |
| (Lai *et al.*, 2021) | Murine study *in vivo* | Midlife hypertension in rats increased cerebral amyloid angiopathy due to Aβ deposition in the somatosensory cortex and cingulate cortex. Hypertension reduced capillary density in these brain cortices and promoted arteriole stiffening. An AD genotype in mice had similar effects on cerebral arterioles. There were no changes in BACE levels or other enzymes involved in Aβ production/degradation. |
| (Gentile *et al.*, 2009) | Murine study *in vivo* | Hypertension was induced in mice through transverse aortic constriction and chronic AngII infusion. This increased the blood-brain barrier permeability in the cortex and hippocampus and thus increased Aβ amyloidosis. Administration of anti-Aβ antibodies reversed these effects. |
| (Kruyer *et al.*, 2015) | Murine study *in vivo* | Mice with AD pathology and chemically induced hypertension showed poorer cognitive performance than wild-type mice with chronic hypertension. There was a greater degree of Aβ cerebral amyloid angiopathy in the AD hypertensive mice compared to the wild-type hypertensive mice. Although, AD mice with hypertension exhibited fewer Aβ plaques in the cerebral parenchyma than wild-type controls, and overall cerebral Aβ burden was similar between groups. Hypertension in the AD mice disrupted tight junctions, predisposed to increased blood-brain barrier permeability, and caused a decline in neuron and pericyte cell populations. |
| (Richardson *et al.*, 2012) | Human autopsy and medical records study in 456 patients | This study investigated the association between self-reported vascular disease and brain pathology. Self-reported stroke was associated with vascular but not AD pathology. Hypertension and heart attach were associated with microinfarction and cerebral amyloid angiopathy. Cerebral small vessel disease and cardiovascular disease are interrelated, but vascular risks were not associated with increased AD pathology. |
| (Lane *et al.*, 2019) | Human observational study in 502 patients | All of the following outcomes were measured when participants reached 69-71 years of age. An increase in systolic and diastolic blood pressure of 10mmHg, each increased white matter hyperintensity volume by 7% and 15% respectively in individuals who were 53 years old. Increases of 10mmHg in systolic blood pressure reduced hippocampal volume by 0.021mL, and an equal increase in diastolic blood pressure reduced whole-brain volume, but not hippocampal volume, by 6.9mL. However, these associations were minimal. Systolic blood pressure above 140mmHg negatively correlated to smaller brain volume and hippocampal volume at 53 years of age. This association did exist at 36-43 years of age but was borderline. Blood pressure did not increase amyloid plaque burden or Preclinical Alzheimer Cognitive Composite scores in 69-71 year olds. |
| (Hu *et al.*, 2022) | Human observational study in 1546 patients | Individuals with hypertension demonstrated lower cognition alongside greater tau biomarker concentrations compared to normotensive individuals. This effect only existed for increases in systolic blood pressure; diastolic blood pressure inversely correlated to CSF levels of tau. Blood pressure did not affect Aβ pathology. |
| (She *et al.*, 2021) | Human observational study in 1069 patients | Individuals with higher systolic and mean arterial blood pressure showed higher blood concentrations of Aβ40 compared to those with normal blood pressure, this effect was only observed in non-carriers of the APOE-ε4 allele. Furthermore, participants with higher blood pressure showed lower Aβ42/Aβ40 ratios. Aβ42 levels were similar between groups. |
| (Janelidze *et al.*, 2016) | Human observational study in 719 patients | Patients with hypertension had increased plasma concentrations of Aβ42 and Aβ40 (both p=0.002). Similar findings were observed in patients with ischaemic heart disease and diabetes. |
| (Petrovitch *et al.*, 2000) | Human observational study in 243 patients | Men in the Honolulu-Asia Aging study who had greater systolic blood pressure (>140 mmHg) demonstrated increased density of NFTs and Aβ plaques compared to normotensive individuals. A higher midlife diastolic blood pressure (>90 mmHg) also associated with greater numbers of hippocampal NFTs. |
| (Shah *et al.*, 2012) | Human observational study in 667 patients | Blood pressure was measured at three examinations from 1965 until 1971, and dementia case finding, and autopsy occurred between 1990 and 2000. As systolic and diastolic blood pressure increased plasma Aβ40 and Aβ42 increased. Lower levels of the Aβ peptides increased the risk of AD and cerebral amyloid angiopathy. Diastolic blood pressure interacted with each Aβ peptide to increase the risk of AD. |
| (Rosano *et al.*, 2024) | Human observational study in 309 patients | Women had significantly lower serum Aβ42/Aβ40 ratios, and higher p-tau and neurofilament light concentrations than men. Greater serum p-tau concentrations associated with hypertension, and lower Aβ42/Aβ40 significantly associated with type 2 diabetes mellitus. |
| (Xu *et al.*, 2023b) | Human observational study in 528 patients | Cross-sectional analysis revealed a relationship between higher systolic blood pressure and poorer cognition overall, as well as poorer episodic memory and language in patients carrying autosomal dominant mutations for AD. Diastolic blood pressure did not associate with cognitive performance. There were no longitudinal associations between blood pressure and cognitive performance in patients with and without AD mutations. |
| (Ruthirakuhan *et al.*, 2024) | Human observational study in 11,074 patients | Patients with hypertension and diabetes mellitus had the greatest risk of AD compared to individuals with neither condition, followed by participants with hypertension alone. The presence of hypertension and AD increased the risk of AD in individuals with underlying cerebral amyloid angiopathy, NFTs, and Aβ plaques. |
| (Ren *et al.*, 2024) | Human observational study in 5432 patients | Using hierarchical cluster analysis, it was shown that hypertension (in a cluster with diabetes, hyperlipidaemia, stroke, chronic kidney disease, and varicose veins) increased the risk of vascular dementia, but not AD. Similarly, ischaemic heart disease, heart failure, and atrial fibrillation (in a cluster with thyroid disease, and musculoskeletal pathology) increased the risk of vascular dementia, but not AD. After Bonferroni correction, associations with either cluster with levels of neurofilament light and Aβ42 became non-significant. |
| (Scambray *et al.*, 2023) | Human observational study in 5136 patients | Hypertension associated with a lower odds of AD neuropathology (odds ratio 0.81) and cerebral amyloid angiopathy (odds ratio 0.79), especially in males and females respectively. Decreased likelihood of cerebral amyloid angiopathy and AD neuropathology associated with cardiovascular events. |
| (Kapasi *et al.*, 2024) | Human observational study in 765 autopsies | Patients with severe atherosclerosis demonstrated a 17% reduction in the mean hippocampal-to-hemisphere volume ratio. Each additional 10mmHg increase in systolic blood pressure attenuated this association by 0.1 units. |
| (Tosto *et al.*, 2016) | Human observational study in 46 patients | Hypertension correlated to a lower risk of late-onset AD (odds ratio 0.63). A history of stroke increased the risk of late-onset AD (odds ratio 2.23). A sample from the WHICAP study, aiming to replicate the results of this study, showed that hypertension, diabetes, or heart disease did not alter the risk of late-onset AD. |
| (Chouraki *et al.*, 2015) | Human observational study in 2189 patients | An increase in plasma Aβ42 by one standard deviation associated with a reduced risk of AD (hazard ratio 0.79) and dementia (hazard ratio 0.80). The plasma ratio of Aβ42/Aβ40 showed a similar association. Aβ40 did not associate with the risk of AD or dementia. |
| Vascular risk scores may be useful in predicting the development of Alzheimer’s disease | | |
| (Han *et al.*, 2024) | Human observational study in 136 patients | Serum low-density lipoprotein concentrations did not associate with Aβ or tau deposition. However, there was an interaction effect between higher concentrations of low-density lipoprotein and cerebral Aβ amyloidosis, especially for individuals with low-density lipoprotein levels of ≥116 mg/dL. No other lipids demonstrated this relationship with Aβ and tau. |
| (Kuo *et al.*, 1998) | Human observational study in 100 autopsies | Serum low-density lipoprotein cholesterol and apolipoprotein-B correlate with greater cerebral Aβ42 amyloidosis. This relationship was not found with Aβ40. |
| (Bennett *et al.*, 2020) | Human observational study in 325 patients | Total cholesterol and low-density lipoprotein cholesterol positively correlated with amyloid burden on positron emission tomography. However, these associations were diminished after accounting for age, race, gender and educational attainment. Triglyceride and high-density lipoprotein levels were not associated with Aβ amyloidosis. |
| (Hughes *et al.*, 2024) | Human observational study in 6814 patients | Many subclinical cardiovascular risk factors were studied including, but not limited to, blood pressure, carotid artery stenosis, carotid artery intima-media thickness, coronary artery calcification, and systemic vascular resistance. The presence of these factors reduced the time-to-event for coronary heart disease and dementia over a follow-up period of 15 years. These results did not vary based on sex or ethnicity. |
| (Lockhart *et al.*, 2022) | Human observational study in 159 patients | Higher Framingham stroke risk profile and cardiovascular risk factor scores correlated to higher cerebral Aβ burden. Vascular risk factor scores also correlated to reduced cortical thickness in the temporal lobe and larger white matter hyperintensities. Evidence of Aβ deposition associated with a decline in cognition over a 6-year period. |
| (Sapkota *et al.*, 2023) | Human observational study in 200 patients from two study cohorts | Higher cerebral Aβ amyloidosis correlated to lower plasma Aβ42/Aβ40. In individuals carrying the APOE ε4 allele, a higher vascular risk score is associated with greater cerebral Aβ deposition. |
| (Kim *et al.*, 2019) | Human observational study in 222 patients | Linear regression analysis revealed that in the presence of vascular risk factors (such as hypertension, diabetes, coronary heart disease, and stroke), cerebral Aβ retention on positron emission tomography is associated with subjective memory impairment. |
| (Ferrari-Souza *et al.*, 2024) | Human observational study in 503 participants | The presence of more vascular risk factors did not significantly associate with AD pathophysiology (p=0.754). Elevated vascular risk factor burden acted alongside preclinical AD to increase neurofilament light concentrations and cognitive decline over time. A poorer cognitive performance at baseline slightly associated with the presence of more vascular risk factors (p=0.063). Vascular risk factors did not associate with CSF concentrations of Aβ42 or p-tau over time. |
| (García-Lluch *et al.*, 2024) | Human observational study in 233 patients | Patients with and without AD pathology and biomarkers were recruited. The ERICE and SCORE2 cardiovascular risk scores were associated with AD pathology, but the Framingham Risk Score (FRS) was not associated with AD. |
| (Shirzadi *et al.*, 2024) | Human observational study in 166 patients | Elderly patients without cognitive impairment were recruited from the Harvard Aging Brain study. Linear regression analysis showed that after adjusting for sex and educational attainment, age did not contribute to cognitive decline, whereas cardiovascular risk, cerebral perfusion, and Aβ/tau amyloidosis did. |
| (Bilgel *et al.*, 2021) | Human observational study in 87 patients | 10-year vascular risk, determined by the FRS, did not correlate to Pittsburgh compound B or 18F-flortaucipir positron emission tomography, indicating cerebral Aβ and p-tau amyloidosis respectively. |
| (Rabin *et al.*, 2018) | Human observational study in 223 patients | A higher FRS did not independently associate with cerebral Aβ amyloidosis (p=0.30) or hippocampal volume (p=0.30), despite associating with white matter hyperintensities (p=0.002). The risk score and Aβ burden were each associated with faster Preclinical Alzheimer cognitive composite decline, and both variables interacted to potentiate the decline in this outcome. Indeed, cognitive decline was accelerated in participants who both tested positive for Aβ on imaging and had higher FRS, compared to Aβ-negative patients. |
| (Rabin *et al.*, 2022) | Human observational study in 196 patients | Elevated vascular risk determined by the FRS and Aβ burden associated with the thinning of the frontal and temporal cortices over time. Vascular risk did not associate with greater atrophy in the parietal or occipital cortices, whereas greater Aβ burden did associated with accelerated atrophy in these regions. |
| (Conner *et al.*, 2019) | Human observational study in 129 patients | A one-point increment in mid-life cardiovascular risk, measured using the Framingham stroke risk score, increased the odds of cortical infarction (odds ratio 3.99), subcortical infarction (odds ratio 1.95), cerebral atherosclerosis score (odds ratio 1.88) and cerebral arteriosclerosis score (odds ratio 1.74). The mid-life vascular risk did not associate with Braak NFT stage, CAA, or CERAD plaque score. In late life, a one-point increase in vascular risk increased the Braak stage by 3%. |
| (Jiang *et al.*, 2023) | Human observational study in 1521 patients | In patients without dementia, those with a higher FRS had lower composite cognitive scores than participants with lower risk scores. After adjusting for confounders such as education and demographics, a higher FRS associated with increased t-tau and neurofilament light, but a higher risk score did not associate with Aβ peptide levels. |
| (Yu *et al.*, 2021) | Human observational study in 491 patients | In patients without cognitive impairment, a higher FRS associated with increased decline in MMSE score and executive functioning. F18-fluorodeoxyglucose uptake on positron emission tomography was lower in patients with higher FRS. |
| (Tranfa *et al.*, 2024) | Human observational study in 606 patients | A patient’s cardiovascular risk, ascertained by the FRS, associated with changes in the direction of diffusion in the fornix and cingulum of the limbic system and the splenium and genu of the corpus callosum, measured by fractional anisotropy and mean diffusivity. Also, higher risk scores associated with changes in diffusion in the corona radiata and internal capsule. |
| (Saeed *et al.*, 2024) | Human observational study in 135 patients | Greater levels of a neurodegeneration biomarker associated with increasing mid-life atherosclerotic cardiovascular risk score (odds ratio 6.98, p<0.05), after a 16-year follow-up. However, this risk score did not associate with levels of Aβ or tau. Late-life white matter hyperintensity lesions correlated with a greater risk score. |
| (Pålhaugen *et al.*, 2021) | Human observational study in 589 patients | Periventricular and deep white matter hyperintensities were greater in the cerebrum in individuals with imaging evidence of Aβ deposition. After adjusting for age, which itself associated with more severe white matter hyperintensities, FRS associated with greater global white matter hyperintensities as well as frontal periventricular and deep hyperintensities. |
| (Yau *et al.*, 2022) | Human observational study in 175 patients | Patients with a higher baseline FRS and elevated Aβ were shown to have greater tau deposition. Of the individual components of the score, a higher systolic blood pressure and body mass index separately interacted with Aβ, resulting in increased tau deposition in the inferior temporal cortex. |
| (Rabin *et al.*, 2019) | Human observational study in 152 patients | In a cohort of patients without cognitive impairment, the FRS did not associate with Aβ amyloidosis (p=0.59), and weakly associated with tauopathy in the inferior temporal cortex (p=0.02), but not tauopathy in the entorhinal cortex (p=0.15). However, FRS and Aβ burden interacted to potentiate tau amyloidosis in the inferior temporal cortex, but not the entorhinal cortex. |
| (James *et al.*, 2023) | Human observational study in 362 patients | FRS in individuals over the age of 70 is associated with normal-appearing white matter with poor structural integrity. Higher blood pressure at midlife correlated to poorer late-life white matter integrity in females only. |
| (Keuss *et al.*, 2022) | Human observational study in 346 patients | In patients without cognitive impairment, a higher FRS at age 69 associated with faster rates of whole-brain atrophy and hippocampal atrophy in individuals without Aβ deposition. However, in patients with evidence of cerebral Aβ amyloidosis, a higher score was associated with slower rates of atrophy. |
| (Lin *et al.*, 2021) | Human observational study in 55 patients | An individual’s vascular risk was assessed using a composite score consisting of 5 variables: hypertension, hypercholesterolemia, diabetes, smoking and body mass index. Elderly participants with mild cognitive impairment demonstrated greater blood-brain barrier breakdown compared to cognitively healthy controls. A greater blood-brain barrier permeability associated with poorer episodic memory, executive function, and scoring on the Montreal cognitive assessment. Furthermore, blood-brain barrier permeability was associated with increased vascular risk scores and lower CSF Aβ42/Aβ40 ratios. |
| (Oveisgharan *et al.*, 2020) | Human observational study in 1585 autopsy specimens | FRS did not associate with AD diagnosis (p=0.766) or levels of Aβ or tau (p=0.918). Using a linear regression model where Aβ, FRS, and their interaction as predictor variables and tau as the outcome variable, it was shown that a lower FRS predisposes to a greater interaction between Aβ and tau. The only component of the risk score to independently affect this interaction was systolic blood pressure, where a lower blood pressure predicted a greater association between Aβ and tau. |
| Platelet activation and thrombosis impact Alzheimer’s biomarker changes | | |
| (Wolska *et al.*, 2023) | *In vitro* study | Hypoxia induced the release of Aβ42 that was stored inside of platelets. Inhibiting BACE1 did not affect platelet Aβ release. |
| (Wolozin *et al.*, 1998) | *In vitro* study | Aβ40 augmented the aggregation of platelets in response to adenosine diphosphate (ADP) by 2-fold. Other Aβ peptides did not have similar effects on platelet aggregation. |
| (Ramos-Cejudo *et al.*, 2022) | Human observational study in 1847 patients | After 20 years of follow-up, patients with higher adenosine diphosphate-mediated platelet aggregation were shown to have a higher risk of all-cause dementia and AD. |
| (Abubaker *et al.*, 2019) | *In vitro* study | Aβ42 increased platelet adhesion compared to scrambled Aβ42; this finding was statistically significant. Aβ40 and Aβ25-35 increased platelet adhesion compared to scrambled Aβ42, this did not reach statistical significance. Aβ peptides induced morphological changes indicative of platelet activation. Platelet adhesion and thrombosis did not occur in response to Aβ peptides at arterial shear stress (1,000/sec) but did occur in response to Aβ42 at venous shear (200/sec). |
| (Donner *et al.*, 2020) | *In vitro* study | Aβ40 requires α_IIb_β_3_ integrins and glycoprotein VI receptors to increase platelet aggregation and release of adenosine triphosphate and fibrinogen. Conversely, cultures of platelets taken from glycoprotein VI-knockout mice showed less Aβ aggregation, and blocking α_IIb_β_3_ receptors further reduced Aβ aggregation. Genetic ablation of the glycoprotein VI receptor in mice led to reduced platelet adhesion in injured carotid arteries in response to Aβ stimulation. |
| (Donner *et al.*, 2024) | *In vitro* study | Platelets from male AD transgenic mice showed greater quantities of dense granules compared to wild-type mice. However, dense granules from AD and wild-type mice showed similar release of serotonin and adenosine triphosphate. There were sex-specific differences in thrombus formation in AD transgenic mice compared to control mice. |
| (Visconte *et al.*, 2018) | *In vitro* study | Compared to wild-type platelets, APP-knockout platelets demonstrated similar adherence to collagen, von Willebrand factor and fibrinogen and similar clot retraction. Aβ40, Aβ42 and Aβ25-35 adhered to platelets, especially Aβ25-35, which is the biologically active sequence of Aβ. When APP was genetically ablated, platelets could not adhere to these peptides. Aβ peptides also bound to HEK293 and MDA-MB-231 cells, which both express APP. |
| (Yang *et al.*, 2021) | Human observational study in 646 patients | Anaemic participants had lower MMSE scores than patients without anaemia, indicating that anaemia confers a degree of cognitive impairment. Patients with anaemia showed lower levels of Aβ42 in the CSF, but other AD biomarkers such as Aβ40 and p-tau were similar between groups. |
| (Kim *et al.*, 2021) | Human observational study in 428 patients | Aβ deposition showed no relationship with haemoglobin level, and haemoglobin level did not independently associate with cognitive impairment. Lower haemoglobin levels were associated with abnormal glucose metabolism in brain regions sensitive to AD pathology such as the posterior cingulate cortex, inferior temporal gyri and angular gyri. |
| Greater arterial stiffness associates with cerebral amyloid angiopathy and Alzheimer’s pathology | | |
| (Moore *et al.*, 2021) | Human observational study in 146 patients | In patients older than 73, pulse wave velocity, used as a measure of aortic stiffness, positively correlated with CSF p-tau, t-tau, and neurogranin and YKL-40, markers of synaptic dysfunction and neuroinflammation, respectively. After excluding individuals with comorbidities such as CVD and atrial fibrillation all associations except that with YKL-40 became non-significant. There was no evidence that pulse wave velocity associated with cerebral Aβ deposition. |
| (Pasha *et al.*, 2020) | Human observational study in 32 patients | After adjusting for confounders, the association between carotid-femoral pulse wave velocity and cerebral Aβ deposition was no longer significant. Carotid β stiffness index positively correlated with mean cortical Aβ uptake after adjusting for confounders. |
| (Hughes *et al.*, 2018) | Human observational study in 321 patients | In this subanalysis of the Atherosclerosis Risk in Communities study, an increase in the arterial stiffness, measured by pulse wave velocity, of the vessels between the heart and common carotid artery by one standard deviation was associated with an increased odds of exhibiting imaging evidence of Aβ by 30%. Furthermore, this increased stiffness associated with smaller brain volumes, white matter hyperintensities, and microhaemorrhages. |
| (Cui *et al.*, 2018) | Human observational study in 356 patients | Carotid-femoral pulse wave velocity significantly associated with hypertension, diabetes, mild cognitive impairment, and cerebral infarction. Higher carotid-femoral pulse wave velocity independently associated with a higher risk of dementia. |
| (Wagner *et al.*, 2022) | *Ex vivo* study, *in vitro* study, and murine study *in vivo* | Medin accumulated alongside Aβ in APP-overexpressing mice. Genetic ablation of medin eliminated Aβ plaque formation and reduced cerebral amyloid angiopathy by 85% compared to wild-type mice. MFG-E8 levels were higher in mice with more severe cerebral amyloid angiopathy. Injection of medin deposits into the murine hippocampus resulted in Aβ aggregation. |
| (Davies *et al.*, 2014) | *In vitro* study | Medin can aggregate to form fibrils over 48 hours, involving a characteristic lag phase and then an exponential growth and elongation phase. Medin comprises a partial β-sheet structure alongside α-helical and random coil elements. |
| (Davies *et al.*, 2015) | *In vitro* study | Medin shares a similar amino acid sequence to Aβ and forms a secondary structure that arranges to form amyloid-like fibrils. Specifically, the Asp(25) and Lys(30) residues of medin align with Asp(23) and Lys(28) of Aβ. Medin self-aggregated into a β-sheet fibril. |
| (Kumar *et al.*, 2020) | Human observational study in 82 patients | In patients at risk of AD due to family history, those of African American origin demonstrated lower t-tau and p-tau levels in their CSF than non-Hispanic White Americans. African American patients had higher central blood pressures and vascular stiffness on ultrasound scanning. Furthermore, African American patients exhibited poorer cognition on cognitive testing than white patients. |
| (Cooper *et al.*, 2022) | Human observational study in 257 patients | Central pulse pressure, forward wave amplitude, and carotid-femoral pulse wave velocity, which are markers of aortic pressure and pressure pulsatility, associated with greater tau amyloidosis in the entorhinal and rhinal areas, especially in patients older than 60. However, carotid-femoral pulse wave velocity did not associate with tauopathy in these regions. Also, no association between these measures and cerebral Aβ load was noted. |
| Amyloid fibrils within in the heart | | |
| Cardiac amyloidosis and the association with Alzheimer’s disease | | |
| (Annamalai *et al.*, 2017) | Human observational study in 2 patients | In patients with systemic light chain amyloidosis, green birefringence on Congo red staining, and light chain fragments were noted on heart specimens. Also, X-ray diffraction patterns due to amyloid deposition are similar in different tissues within the same patient but show interpatient variability. |
| (Nguyen *et al.*, 2024a) | Human observational study in 1 patient | ATTR fibrils from a patient carrying the V30M transthyretin mutation showed a similar ‘closed gate’ structure in the heart and nerves. This structure consists of N-terminal and C-terminal fragments enclosing a polar channel. |
| (Uneus *et al.*, 2022) | Human observational study in 18 patients | 55% of patients with ATTR amyloidosis due to the V30M mutation showed pathological cerebral amyloidosis; patients with AD showed rates of 100%. 60% of patients with ATTR showed pathological amyloidosis in the cerebellum, compared to 20% of patients with AD. |
| (Nguyen *et al.*, 2024c) | Human observational study in 5 patients | Transthyretin fibrils were morphologically distinct between familial ATTR patients carrying I84S or V30M mutations and wild-type ATTR patients. |
| (Nguyen *et al.*, 2024b) | Human observational study in 4 patients. | Wild-type ATTR fibrils demonstrated a similar structure in human cardiomyopathies. |
| (Fernandez-Ramirez *et al.*, 2024) | Human observational study in 3 patients | Cardiac specimens from three patients carrying the T60A mutation in transthyretin were analysed. Fibril structure was similar between all patients and similar to fibril structure in other organs. |
| (Levites *et al.*, 2024) | Murine study *in vivo* and *in vitro* study | Pleiotrophin and midkine are found in senile plaques alongside Aβ and potentiate amyloidosis in CRND8 mouse models of AD, and in brain specimens from AD patients. Pleiotrophin and midkine also accumulate with transthyretin in the heart. |
| (King & Robinson, 2020) | *In vitro* study | In 14 heart samples, 28S ribosomal protein S5, 26S proteasome non-ATPase regulatory subunit 11, and mitochondrial proteins were upregulated in mice with AD. In heart and liver specimens, immunoglobulins were downregulated in AD models. Proteins involved in the mitochondrial electron transport chain were increased in hearts from AD models. |
| (Hendren *et al.*, 2024) | Human observational study in 302 patients | V122I carriers had lower circulating transthyretin than non-carriers. In the first study phase, transthyretin negatively correlated to left ventricular ejection fraction. In the second phase, V122I carriers had thicker left ventricles and transthyretin levels negatively correlated to left atrial volume. |
| (Hamasaki *et al.*, 2022) | Human observational study in 240 patients | ATTR deposition positively correlated with Aβ and NFT burden (measured by Braak score) in patients <90 years old, and with the presence of heart failure. No correlation existed between cardiac fibrosis and cerebral NFTs. |
| (Heuschkel *et al.*, 2020) | Observational study in 11 valve donor specimens | Calcified regions of aortic valves stained positive for transthyretin protein, APP, and Aβ. Non-calcified regions and valves from healthy donors did not stain from these proteins. |
| (Mielcarek *et al.*, 2014) | Murine study *in vivo* | Cardiac huntingtin amyloidosis in mice caused bradycardia, and elongation of PQ, QT, ST, QTS, and RR intervals with higher HRV. Left ventricular end-diastolic volume and cardiac output were reduced, and genes involved with cardiac fibrosis were upregulated. |
| The role of pre-amyloid oligomers on the heart | | |
| (Sidorova *et al.*, 2015) | *In vitro* study | Rapid pacing (termed ‘rapid activation’) of the cultured atrial cell line, HL-1, increases pre-amyloid oligomer formation and reactive oxygen species production, the latter produces γ-ketoaldehydes which may potentiate amyloidosis. |
| (Rainer *et al.*, 2018) | Murine study *in vivo* and human observational study | Cardiac hypertrophy and heart failure associated with increased pre-amyloid oligomer formation and desmin cleavage compared to healthy controls. Desmin phosphorylation at Ser31 is responsible for its accumulation. Cleavage of the pre-amyloid oligomer desmin is increased in human cardiomyopathies. |
| (Sanbe *et al.*, 2005) | Murine study *in vivo* and *in vitro* study | Overexpressing of the R120G mutation in the α-B-crystallin protein results in increased cardiac amyloidosis, cardiomyocyte toxicity, and hypertrophy compared to wild-type mice, as well as reduced fractional shortening and heart failure. |
| Amyloid β and the cardiovascular system | | |
| Amyloid β peptides negatively affect cardiomyocyte physiology | | |
| (Arai *et al.*, 1991) | Human observational study in 30 autopsies | APP was shown to be present in the myocardium, aorta, pituitary gland, and adrenal gland at autopsy. |
| (Troncone *et al.*, 2016) | Human observational study in 57 patients and *in vitro* study | Patients were divided into three age categories: <65, 65-80, and >80. Diastolic dysfunction, measured as the mitral valve E/A ratio, was present in patients <65 years old (1.20±0.18 for AD patients compared to 1.34±0.12 for controls), but not in the other age categories. Left ventricular septal wall thickness was increased in AD patients compared to controls in the >80 years category only (1.12±0.05 vs 1.01±0.04, p<0.05), as was the left ventricle inferolateral wall thickness (1.07±0.05 vs 1.00±0.03, p<0.05). The presence of Aβ was confirmed using transmission electron microscopy and anti-Aβ antibodies. |
| (Ohgami *et al.*, 1993) | *In vitro* study | L-beta A4 APP, a splicing product of the APP gene excluding exon 15, was found in the brain, spinal cord, heart, and other tissues of Sprague-Dawley rats. |
| (Skodras *et al.*, 1993) | Human observational study in 18 autopsies | Whilst plaques were found in the hippocampus of specimens from AD patients, no plaques were visualised in the hearts of these patients. |
| (Hart *et al.*, 2001) | Murine study *in vivo* and *in vitro* study | Transgenic mice overexpressing the heparin sulphate proteoglycan, perlecan, were studied. Perlecan messenger RNA was upregulated two-fold in the hearts of transgenic mice compared to the wild-type mice (p<0.05), and four-fold in the brains of transgenic mice. |
| (Kheirbakhsh *et al.*, 2018) | Murine study *in vivo* | Injection of Aβ42 into rat hippocampi induced elevations of triglyceride, glucose, and insulin compared to control rats, indicating a degree of insulin resistance. Hepatic and renal amyloid bodies were observed, and pathological blood vessel congestion was noted. |
| (Jang *et al.*, 2022) | *In vitro* study | Aβ40 and Aβ42 reduced endothelial cell and cardiomyocyte viability, decreased inner mitochondrial membrane potential (ΔΨm), and increased Ca^2+^-induced mitochondrial swelling by opening mitochondrial pores, compared to controls. Aβ42 incubation increased reactive oxygen species production. |
| (Ferreira *et al.*, 2015) | *In vitro* study | Aβ increased cellular [Ca^2+^] and decreased ΔΨm compared to controls. |
| (Turdi *et al.*, 2009) | Murine study *in vivo* and *in vitro* study | APPswe/PS1de9 mice showed higher heart rates and changes in the morphology of the RS complex of the ECG relative to wild-type littermates. There was no histological evidence of interstitial fibrosis. Experiments with isolated cardiomyocytes indicated a loss of contractility of APPswe/PS1de9 cardiomyocytes relative to wild-type controls associated with a reduced Ca^2+^ transient amplitude, and a loss of sensitivity to adrenergic agonists. |
| (Jang *et al.*, 2023) | *In vitro* study | In cardiomyocytes and endothelial cells, Aβ42 reduced amino acids and fatty acid levels and increased lipid peroxidation. Aβ42 and Aβ40 reduced respiration rate in both cell types. |
| (Sakamuri *et al.*, 2022) | *In vitro* study | Aβ42 impaired respiration in human brain microvascular endothelial cells and mitochondrial adenosine triphosphate production. This impairment is more severe in hypoglycaemic conditions. Also, osmolarity and endothelial cell senescence alter the effects of Aβ on mitochondrial metabolism. |
| (Schmidt *et al.*, 2008) | *In vitro* study | APP and Aβ bound to the adenosine triphosphate synthase complex from a mitochondrial fraction of mouse heart specimens. Incubation of a human fibroblast fraction containing ATP synthase complex demonstrated that APP inhibited ATPase activity. Additionally, APP and Aβ both reduced ATP production by up to 30% in C6 astrocytoma cells, which contain the ATP synthase complex. |
| (Murphy *et al.*, 2022) | *In vitro* study and murine study *in vivo* | 5xFAD mice showed a reduction in fractional shortening compared to wild-type mice, progressing until 6 months of age. This occurred alongside a longitudinal reduction in ejection fraction. Electrocardiography in 5xFAD mice revealed a reduced amplitude of P, R, and T waves in lead II. Cardiomyocytes from 5xFAD mice showed a reduction in sarcomere shortening compared to wild-type cardiomyocytes and decreased calcium flux. 5xFAD mice exhibited a reduction in cardiac and cerebral mitochondrial oxidative phosphorylation and adenosine triphosphate production. |
| (Hall *et al.*, 2024) | Murine study *in vivo* | BACE1 and PS1 were increased in adipose tissue of obese mice. Aβ42 administration reduced myocardial glucose clearance, increased lipid formation, and impaired diastolic function. Aβ42 increased cardiomyocyte ANP expression. |
| (Tang *et al.*, 2023) | *In vitro* study and *in vivo* worm study | Atrial natriuretic peptide (ANP) was shown to reduce Aβ aggregation into plaques *in vitro*. Cross-seeding ANP with Aβ in worms reduced paralysis induced by Aβ peptides by up to 34.4%. Reactive oxygen species formation in the worms was reduced after the addition of ANP by up to 50.5% compared to worms not given ANP. |
| (Zhang *et al.*, 2014) | *In vitro* cellular study | Aβ25-35 resulted in complete apoptosis in 46.1% of cardiomyocytes, with 15.68% entering early apoptosis. Aβ25-35 increased markers of endoplasmic reticulum stress and cytoskeleton damage. |
| (Botteri *et al.*, 2018) | *In vitro* study and murine study *in vivo* | Palmitate increased the levels of BACE1 and induced endoplasmic reticulum stress and the release of stress markers including sXbp1, Atf3, Chop, and Bip from mouse C2C12 myoblasts. Inhibiting BACE1 using Merck-3 ameliorated the stress and the rise in these markers, and this inhibition reduced the release of inflammatory IL-6 and tumour necrosis factor-α. BACE1 inhibition also prevented the fall in Pgc-1α, a gene involved in regulating fatty acid oxidation, induced by palmitate. sAPPβ, produced by BACE1-mediated APP cleavage, mimicked the effects of palmitate, and reduced levels of mitochondrial complexes I, II, III, IV, and V. Administering sAPPβ to mice *in vivo* has the same effects as *in vitro*. |
| (Song *et al.*, 2008) | Murine study *in vivo* and *in vitro* study | Aβ increased the expression of E2-25K/Hip-2. This complex stabilises the caspase-12 protein via proteasome inhibition, which then contributes to Aβ-mediated neurotoxicity. |
| (Anwar & Mabrouk, 2023) | Murine study *in vivo* and *in vitro* study | Neuroinflammation in rats associated with increased cerebral Aβ and tau deposition. A positive correlation was seen between Aβ and troponin I release. Histologically, cardiac muscle striation was disturbed, alongside intrafibrillar haemorrhage, inflammatory cell infiltration, and necrosis versus controls. |
| (Haase *et al.*, 2013) | *In vitro* study and *ex vivo* study | Aβ25-35 had a positive chronotropic effect on cardiomyocytes via α1-adrenoceptors and increased aortic and coronary artery vasoconstriction. Aβ1-15, Aβ40, and Aβ42 had negative chronotropic effects. |
| (Krämer *et al.*, 2018) | *In vitro* study and human observational autopsy study | Myocardial-specific inclusions containing sAPPδ and sAPPη were found mostly outside the conduction system. These inclusions associated with myocardial fibrosis in autopsy specimens and cerebral amyloid angiopathy but not cerebral Aβ or NFT burden in patients. |
| (Elia *et al.*, 2023) | Murine study *in vivo* | Left ventricular ejection fraction decreased and left ventricular end-systolic volume increased in Tg2576 mice compared to wild-type mice. Tg2576 mice showed increased myocardial fibrosis and cardiac Aβ40. Cerebral and cardiac brain-derived neurotrophic factor levels declined which coincided with a loss of sympathetic cardiac nerve fibre density. |
| Amyloid β alters nitric oxide physiology and endothelial function | | |
| (Austin & Katusic, 2020) | Murine study *in vivo* | Heterozygous mice lacking one allele for functional endothelial NOS (eNOS^+/-^) mice showed increased microvascular Aβ40 and PS1 expression compared to wild-type mice. |
| (Cifuentes *et al.*, 2017) | Murine study *in vivo* | Inhibition of eNOS resulted in cognitive impairment in APPswe/PS1de9 mice and predisposed to increased cerebral Aβ42 deposition and cerebral amyloid angiopathy than wild-type mice. |
| (Austin & Katusic, 2016) | Murine study *in vivo* and *ex vivo* study | Cerebral levels of p25, an activator of the tau kinase Cdk5, were higher in APPswe/PS1de9 mice deficient in eNOS compared to wild-type mice or APPswe/PS1de9 mice not deficient in the enzyme. The ratio of p25/p35, an indicator of increased Cdk5 activity, was greater in transgenic mice compared to wild-type mice. This translated to greater cerebral levels of p-tau in the endothelial nitric oxide synthase knockout mice compared to the other models, including in the hippocampus. There was no histological evidence of NFTs in brain sections from the mouse models |
| (Oliveira *et al.*, 2011) | In vitro study | Aβ42 induced death in 82% of retinal neurons versus controls. Nitric oxide production was decreased by 34% after Aβ incubation. |
| (Lamoke *et al.*, 2015) | *In vitro* study | In endothelial cells, Aβ42 and Aβ25-35 reduced eNOS levels and protein kinase B phosphorylation. Aβ42 and Aβ25-35 increased reactive oxygen species production. |
| (Parodi-Rullan *et al.*, 2020) | *In vitro* study | Aβ fragments that do not aggregate, such as Aβ16 and Aβ34, did not induce apoptosis of cerebral microvascular endothelial cells. Aβ42 did induce apoptosis of endothelial cells after 1 and 3 days of incubation. Aβ42, and to a lesser extent Aβ40, inhibited angiogenesis. |
| (Chisari *et al.*, 2010) | *In vitro* study | Incubation of rat arteries with Aβ40 or Aβ25-35 increased vasoconstriction and reduced vasodilation, and Aβ25-35 reduced endothelial NOS phosphorylation. |
| (Price *et al.*, 2001) | *In vitro* study | Aβ40 and Aβ25-35 peptides reduced vasodilation and reduced nitric oxide production and were toxic to endothelial cells. |
| (Sutton *et al.*, 1997) | *In vitro* study | Aβ induced endothelial cell necrosis and mitochondrial swelling. Aβ significantly reduced nitric oxide production but did not affect levels of NOS. |
| (Suo *et al.*, 1997) | *In vitro* study | Endothelial cells underwent apoptosis and necrosis after incubation with Aβ42 or Aβ25-35, mediated by oxidative stress and a rise in intracellular Ca^2+^. |
| (Carelli-Alinovi *et al.*, 2016) | *In vitro* study | Aβ40 reduced nitric oxide production alongside reduced endothelial nitric oxide synthase activity via inhibition of acetylcholinesterase. |
| (Niwa *et al.*, 2001) | *In vitro* study | Aβ40 reduced cerebral blood flow and enhanced the vasoconstriction response due to a thromboxane A2 analogue. Administration of superoxide dismutase or another free radical scavenger prevented this effect. |
| (Soriano *et al.*, 2003) | *In vitro* study | Aβ42 and Aβ25-35 reduced the production of formazan by endothelial cells, on MTT assay, perhaps indicating a reduction in the cells’ mitochondrial metabolic activity. Administering antioxidants to the cells incubated with Aβ did not affect this result. Furthermore, treating the cells with a lysosomal proton influx inhibitor prevented the effects seen on the MTT assay. In addition, it was shown using lactate dehydrogenase and Neutral Red assays that high-dose Aβ peptides did not affect endothelial cell viability. |
| (Singh Angom *et al.*, 2019) | *In vitro* study | Endothelial cells became senescent after incubation with Aβ42 shown by increased β-galactosidase staining and increased p21 and p53 levels. VEGFR-1 protein levels increased after Aβ42 administration, and VEGFR-1 upregulation causes endothelial cells to become senescent through the p21/p53 pathway. |
| (Sun *et al.*, 2018) | *In vitro* study | There was upregulation of BACE1 in senescent human brain microvascular endothelial cells as well as greater levels of Aβ40 peptide. Inhibiting BACE1 reduced levels of Aβ40 in the endothelial cells. Angiotensin II increased BACE1 activity in senescent endothelial cells via angiotensin II type 2 receptors and nuclear factor κB activity, as inhibiting these proteins reversed the upregulation of BACE1. |
| (Vellecco *et al.*, 2023) | Murine study *in vivo* | Intracerebroventricular injection of Aβ42 (a murine model of AD) induced a rise in inflammatory factors such as intercellular adhesion molecule-1 and interleukins-6, -17, and -1β. In AD there was an upregulation in interleukin-17 and its receptors in the aortas of mice, which were reduced by administering an antibody against interleukin-17. In these mice, there was a reduction in acetylcholine-induced vasodilation compared to control mice which was reversed upon administration of the interleukin-17 antibody, independent of nitric oxide synthase pathway. Intracerebroventricular injection of Aβ42 also increased platelet aggregation in response to adenosine diphosphate and increased fibrinogen and CD62P levels. The increase in fibrinogen and CD62P were reversed by the administration of the interleukin-17 antibody. |
| (Park *et al.*, 2005) | Murine study *in vivo* | Aβ40 induced reactive oxygen species formation mediated via NADPH oxidase. This oxidative stress is responsible for NO depletion. |
| (Thomas *et al.*, 1997) | *In vitro* study | Aβ enhanced vasoconstriction and reduced vasodilation. Superoxide dismutase administration reversed this effect. |
| (Khalil *et al.*, 2002) | Murine study *in vivo* | Aβ40 reduced vasodilation due to a direct effect on the endothelium and enhanced vasoconstriction through oxidative stress and endothelin-1 production. |
| (Jang & Surh, 2003) | *In vitro* study | Resveratrol ameliorated the cytotoxicity of Aβ25-35 and Aβ42 on PC12 cells. Also, Aβ25-35 increased the proportion of TUNEL-positive cells, and administration of resveratrol reduced this proportion, indicating a preventative effect of resveratrol on apoptosis. Also, the ΔΨm depolarisation and reactive oxygen species accumulation induced by Aβ25-35 were prevented by resveratrol. |
| (Stepanichev *et al.*, 2008) | Murine study *in vivo* | Aβ25-35 increased nitric oxide synthase activity in the cerebral cortex and hippocampus. |
| (Palmer *et al.*, 2012) | Human observational study in 40 autopsies | Brain specimens from patients with AD showed significantly upregulated levels of endothelin-1 compared to healthy brain tissue. Incubation of SH-SY5Y neuroblastoma cells with Aβ42 resulted in a 1.7-fold increase in endothelin-1. |
| (Palmer *et al.*, 2013) | Human post-mortem study and *in vitro* study | Endothelin-converting enzyme-1 activity and endothelin-1 levels are elevated in brain vessels from AD patients but not in vascular dementia. In cultured human brain endothelial cells, both Aβ40 and Aβ42 caused increase of endothelin-1 release (which was more pronounced with Aβ40), and this was alleviated in the presence of antioxidants. No effects on the myocardium were studied. |
| (Palmer *et al.*, 2020) | Murine study *in vivo* | Aβ infusion resulted in hypertension in Wistar rat models, which was prevented by Zibotentan administration. Zibotentan reduced mean blood pressure by 5.90mmHg (p<0.0001). Blood flow to the brain through the carotid artery was unaffected by Zibotentan administration. Aβ infusion had no effect on the very low frequency, low frequency, and high frequency components of HRV, but did increase the very low frequency component of systolic blood pressure variability. |
| (Hung *et al.*, 2015) | Murine study *in vivo* | Mice overexpressing astrocytic endothelin-1 exhibited sensorimotor impairment after cerebral ischaemia and reperfusion, induced by transient middle cerebral artery occlusion. Endothelin-1 overexpression led to greater cerebral oedema, hemispheric degeneration and brain ventricular enlargement after artery occlusion. Tumour necrosis factor-α, cleaved caspase 3, and markers of oxidative stress such as GFAP were elevated in the hippocampus after endothelin-1 overexpression. Furthermore, endothelin-1 potentiated cerebral amyloidosis. |
| (Keil *et al.*, 2004a) | *In vitro* study | Cells overexpressing Swedish mutation APP showed increased nitric oxide, which impaired mitochondrial respiration, and decreased ΔΨm versus wild-type APP or controls, which were reversed with γ-secretase inhibition. |
| (Keil *et al.*, 2004b) | *In vitro* study | In APP over-expressing cells, increased nitric oxide production, which inhibited cytochrome C activity, and reduced adenosine triphosphate production in mitochondria compared to control cells. |
| (Hayashi *et al.*, 2012) | *In vitro* study and murine study *in vivo* | Hypoxia-inducible gene 1 was expressed at great levels in the brain and heart, co-localised with a mitochondrial marker, and associated with numerous components of γ-secretase on the mitochondrial membrane. Hypoxia-inducible gene 1 was shown to reduce the activity of γ-secretase in the mitochondria; neuronal γ-secretase is normally activated during hypoxia. Inhibition of γ-secretase by this gene also reduced Aβ40 production inside the mitochondria, and to a lesser extent Aβ42, and prevented mitochondrial dysfunction due to these peptides. |
| (Solesio *et al.*, 2018) | *In vitro* study | Aβ40 and Aβ42 induced depolarisation of the mitochondrial membranes of neuronal and endothelial cells, which is known to precede cell apoptosis, and increased reactive oxygen species production. Inhibiting carbonic anhydrase prevented loss of the mitochondrial membrane potential, reduced reactive oxygen species production and prevented apoptosis of cells induced by Aβ. Carbonic anhydrase inhibition did not affect adenosine triphosphate production. |
| (de Montgolfier *et al.*, 2019) | Murine study *in vivo* | Transverse aortic constriction increased carotid artery systolic blood pressure in wild-type and APPswe/PS1de9 mice and resulted in cerebral hypoperfusion and microhaemorrhages. Morris Water maze performance was poorer in APPswe/PS1de9 mice who underwent the procedure compared to wild-type mice. Vasodilatory response to acetylcholine was reduced in the APPswe/PS1de9 mice compared to wild-type mice and reduced even further after transverse aortic constriction. The function of nitric oxide synthase was unaffected. Blood-brain barrier permeability increased after the procedure in both models, but more so in the APPswe/PS1de9 mice. The greater neuroinflammation seen in APPswe/PS1de9 compared to wild-type mice was further increased by transverse aortic constriction. Cerebral Aβ amyloidosis was increased in APPswe/PS1de9 mice after the procedure. |
| (Suo *et al.*, 2000) | Murine study *in vivo* | In hypotensive rats, infusion of Aβ40 increased mean arterial blood pressure. Also, it was shown to constrict cerebral vessels in rats with normal blood pressure and reduce cerebral blood flow but did not affect blood pressure values in these rats. However, peripheral blood pressure was not altered, suggesting a limited effect of Aβ on peripheral vasoconstriction. |
| (Taylor *et al.*, 2022) | *In vitro* study | Cerebral arteries from APP23 mice and wild-type mice were dissected and endothelial cells were studied. When external K+ concentrations were altered, APP23 arteries exhibited less vasodilation compared to wild-type mice. This was shown to be due to dysfunctional endothelial cell Kir2.1 channels in APP23 mice. |
| (Peters *et al.*, 2022) | *Ex vivo* study | Aβ40 reduces Ca2+ entry into endothelial cells via NMDA receptors and the frequency of intracellular Ca2+ transients. Cerebral amyloid angiopathy due to Aβ40 deposition inhibited vasodilation of cerebral arteries in 5xFAD mice, one murine model of AD. |
| (Meakin *et al.*, 2020) | Murine study *in vivo* and human observational study in 115 patients | Aortas from obese mice demonstrated reduced phosphorylated endothelial nitric oxide synthase through reducing the actions of protein kinase B and AMPK. There were no differences in aortic lipid deposition, inflammatory cell infiltration, endothelial cell dysfunction or intimal thickening between groups, each characteristic of atherosclerosis. |
| (Khalil *et al.*, 2007) | Human observational study in 337 patients | Patients with AD showed significantly reduced vasodilation in response to acetylcholine compared to saline (expressed as an E/S ratio) than other dementias and controls. Patients with MMSE scores >27 showed higher E/S versus those with MMSE ≤26. |
| (Kitazume *et al.*, 2012) | Human observational study in 56 patients, a murine study *in vivo* and an *in vitro* study | Inflammatory endothelial cells and stimulated platelets released APP770. Plasma APP770 were increased in patients with acute coronary syndrome, but CSF APP770 did not correlate to the degree of cognitive impairment in AD. Coronary ligation to induce myocardial infarction in rats caused an increase in sAPPα before troponin-I increased. |
| Amyloid β peptides predispose to atherosclerosis and heart failure | | |
| (Stamatelopoulos *et al.*, 2015) | Human observational study in 1464 patients | There was a positive correlation between Aβ40 levels and death from cardiovascular disease. Aβ40 positively correlated to pulse wave velocity and was associated with increased carotid artery intima-media thickness and the number of atherosclerotic plaques in peripheral arteries. |
| (Yousefirad *et al.*, 2016b) | *Ex vivo* study | Aβ22-35 negatively associated with left ventricular developed pressure and maximal rats of pressure development of left ventricle. Aβ22-35 did not affect the action potential duration at 90% repolarisation, no chronotropic effect and no effect on coronary perfusion. |
| (Yousefirad *et al.*, 2016a) | *Ex vivo* study | Aβ42 reduced the left ventricular developed pressure, and maximal rate of pressure development of the left ventricle and had negative chronotropic effects on rat hearts. Coronary perfusion was reduced with higher doses of Aβ42 (10 and 100nmol/l). This peptide also increased the monophasic action potential duration at 90% repolarisation. |
| (Aishwarya *et al.*, 2024) | Murine study *in vivo* and *in vitro* study | Atrial contraction was reduced in APPswe/PS1de9 mice, leading to less filling during diastole. Left ventricular dyssynchrony was observed in APPswe/PS1de9 mice. Aβ plaques and amyloid oligomers were observed in the left ventricles and cardiomyocytes of APPswe/PS1de9 mice, and these mice exhibited increased cardiac collagen deposition and fibrosis. APPswe/PS1de9 mice showed lower catalase and superoxide dismutase levels than wild-type mice. There were decreases in the activity of mitochondrial electron transport complexes in the APPswe/PS1de9 mice. |
| (Vetrano *et al.*, 2016) | Human observational study in 48 patients | 61% of patients had left ventricular diastolic dysfunction, shown by an E/E_m_ ratio of greater than or equal to 8. Participants with ventricular dysfunction showed cognitive impairment compared to those with normal diastolic function shown by lower scores on the Wechsler Adult-Intelligence-Scale verbal and performance scores (p=0.003 and 0.007, respectively) and lower Raven’s matrices (11.1±1.1 vs 16.9±1.4). |
| (Jin *et al.*, 2017) | Human observational study in 68 patients | AD patients demonstrated lower ejection fraction values than patients without cognitive impairment (64.50±4.32% compared to 67.59±4.79%, p=0.026). Cardiac chamber dimensions on echocardiography were similar between groups. Patients with AD also showed slower cerebral blood flow velocity, but there was no correlation between this and ejection fraction. |
| (Zhu *et al.*, 2023) | Human observational study in 4156 patients | Aβ40 negatively correlated with left ventricular ejection fraction, but positively correlated with left ventricle mass. Aβ40 did not affect left atrial diameter or cardiac output. A 1-standard deviation increase in Aβ40 increased heart failure risk by 31% in men. Aβ42 alone had no effect on heart failure risk or echocardiographic parameters. |
| (Koemans *et al.*, 2024) | Human observational study in 6120 patients after autopsy | Men had a lower likelihood of Aβ deposition in the cerebral cortex than women (odds ratio 0.68) independent of other factors, cerebral amyloid angiopathy did not differ based on sex. |
| (Sanna *et al.*, 2019) | Human observational study in 66 patients | AD patients showed no evidence of arrhythmias. However, more low-voltage QRS complexes were present in AD patients compared to controls. Diastolic function, measured by E/A ratio, was significantly lower in AD patients than in controls. |
| (Yasojima *et al.*, 2001) | Human autopsy study in 15 specimens | Messenger RNA for APP, PS1 and BACE were visualised in the hearts of patients who had AD, but the levels were not significantly different to the hearts of patients without AD. The levels of neprilysin, which degrades Aβ, were increased in the peripheral compared to the brain in autopsy specimens. |
| (Rossner *et al.*, 2001) | *In vitro* study | Western blotting revealed the expression of BACE in the hearts of transgenic mice but at lower concentrations than in the brain. |
| (Greco *et al.*, 2017) | Murine study *in vivo*, and *in vitro* study | Antisense BACE1 was upregulated two-fold in heart samples from patients with heart failure than controls. Aβ40, but not Aβ42, levels were significantly higher in heart failure specimens. |
| (Plucińska *et al.*, 2016) | Murine study *in vivo* | BACE1 knock-in in a murine model displayed elevated glucose levels, glucose intolerance, and hyperinsulinaemia compared to wild-type mice. Hepatic glycogen levels were reduced in transgenic mice, whereas triacylglycerol content was increased in the liver. Transgenic mice exhibited cerebral hypometabolism of glucose, but cardiac glucose metabolism was unaffected. There was upregulation of the insulin receptor in transgenic mice, indicating a degree of insulin resistance. The ability of the hypothalamus to suppress appetite was increased in the transgenic mice, shown by increased pro-opiomelanocortin expression. Endoplasmic reticulum stress was confirmed in the hypothalamus through a rise in Chop transcription in transgenic mice. |
| Amyloid β amyloidosis has an effect on cardiac arrhythmias | | |
| (Du *et al.*, 2022) | Human observational study in 260 patients | T-tau and Aβ42 were higher in neuronal-derived exosomes from patients with atrial fibrillation compared to healthy controls. Furthermore, interleukin-6 and matrix metalloproteinase-9 were higher in serum from patients with atrial fibrillation compared to healthy controls. Patients with atrial fibrillation also demonstrated greater levels of cognitive impairment than control patients. Atrial fibrillation patients taking anticoagulants demonstrated lower levels of AD biomarkers and inflammatory cytokines than healthy controls. |
| (Johansen *et al.*, 2020) | Human observational study in 316 patients | Atrial cardiopathy was defined as the presence of ≥1 of P-wave terminal force >5000µV x ms in lead 1, serum N-terminal pro-b-type natriuretic peptide >250pg/mL or left atrial volume index ≥34mL/m^2^. Patients with atrial cardiopathy as a composite or left atrial volume index ≥34mL/m^2^ only showed increased odds of florbetapir standardised uptake values >1.2. |
| (Johansen *et al.*, 2022) | Human observational study in 330 patients | Atrial fibrillation did not correlate to cerebral amyloidosis on PET. Florbetapir standardised uptake values did not associate with atrial tachycardia. With every 1% increase in the time a patient had premature atrial contractions, there was a trend towards increased odds of standardised uptake values >1.2. |
| (Agsten *et al.*, 2015) | *In vitro* study | BACE1 was present in cardiomyocytes. BACE1 interacts with KCNQ1 voltage-gated K^+^ channel subunits to reduce the slow component of the cardiac delayed rectifier K^+^ current (I_Ks_). These effects were reproduced in human and mouse atrial cardiomyocytes. |
| (Kong *et al.*, 2013) | *In vitro* study | In SH-SY5Y cells overexpressing APP, nicorandil reduced apoptosis measured by an MTT assay and annexin V/propidium iodide staining. Nicorandil upregulated Bcl-2 and downregulated caspase-3 and Bax in these cells. Nicorandil also increased phosphorylated Akt. |
| (Sachse *et al.*, 2013) | *In vitro* study | γ-secretase and BACE1 cleave KCNE1 and KCNE2 subunits of voltage-gated K+ channels and subunits of Na+ channels, affecting the membrane potential of neurons. Elevated BACE1 activity shifts the activation curve of cardiac KCNQ1/KCNE1 channels to more depolarised potentials. BACE1 overexpression reduces cardiac repolarisation which may be pro-arrhythmogenic. |
| (Timmers *et al.*, 2018) | Placebo-controlled randomised clinical trial in 64 patients | Increasing doses of the BACE1 inhibitor JNJ-54861911 were administered to elderly participants without electrocardiographic abnormalities and a 4-way crossover thorough QT study performed. Increasing doses of JNJ-54861911 in the therapeutic range up to 50mg did not result in QT prolongation. However, at a supratherapeutic dose (150mg) the corrected QT (QTcF) interval was increased by 15.5 milliseconds after 1.5 hours. |
| (Vormfelde *et al.*, 2020) | Human observational study in 372 patients | The BACE1 inhibitor, umibecestat, at a dose of 300mg did not prolong the QTcF interval recorded using Holter and standard 12-lead electrocardiograms. QTcF interval values above 450ms were seen in 7.4% of patients taking placebo and 6.9% of patients on umibecestat. There was no effect of umibecestat on heart rate, PR interval or QRS interval. |
| (Mao *et al.*, 2023) | Human cross-sectional study in 5153 patients | Prolonged rate-corrected QT and JT intervals associated with all cause dementia (AD and vascular dementia) compared to cognitively healthy individuals. JT prolongation correlated to higher concentrations of plasma Aβ40. Patients with AD had higher resting heart rates on average, were female and had more coronary heart disease. Left axis deviation was associated with an increased probability of a patient having dementia, especially vascular dementia. |
| Tau protein and the cardiovascular system | | |
| (Luciani *et al.*, 2023) | Human observational study from autopsy specimens, murine study *in vivo*, and *in vitro* study | T-tau expression was higher in the hearts of AD patients only. P-tau was significantly higher in dilated cardiomyopathy hearts, and AD hearts, versus controls. In mice, tauopathy impaired multiple echocardiographic parameters. *In vitro*, tauopathy induced microtubule dysfunction and post-translational tyrosination. |
| Alzheimer’s disease genes and cardiac physiology | | |
| (Dave *et al.*, 2023b) | Murine study *in vivo* and *in vitro* study | Triple transgenic AD mice had increased heart weight, expression of Col1a1, Myh7 and Nppa genes and cerebral Aβ42 and p-tau amyloidosis than wild-type mice. |
| (Zhu *et al.*, 2022b) | Murine study *in vivo* and *in vitro* study | APPswe/PS1de9 mouse cardiomyocytes showed decreased ΔΨm, mitochondrial number, peak shortening, and maximal velocity of shortening and re-lengthening. On echocardiography, increased left ventricular end-systolic diameter and decreased fractional shortening were observed. These transgenic mice demonstrated increased interstitial fibrosis. Insertion of mitochondrial aldehyde dehydrogenase transgene reversed these effects. |
| (Zheng *et al.*, 2019) | *In* *vitro* study | The metabolome of the liver and kidney were altered in APPswe/PS1de9 mice compared to wild-type mice, but heart metabolomics was unaffected. No cardiac metabolites varied between the two models up to 10 months of age. At 10 months, inosine monophosphate levels were higher in the hearts of APPswe/PS1de9 mice than in wild-type mice. |
| (Murakami & Lacayo, 2022) | Bioinformatics analysis of open-source pathway databases | AD genes were found to be associated not only with AD but also with other common age-related diseases. Among top diseases associated with AD, cardiovascular pathology (including myocardial infarction, heart disease, hypertension, cardiovascular system disease, and vascular disease) was identified to be most common after neurological disorders and type 2 diabetes mellitus. |
| (Wen *et al.*, 2022) | Genetic analysis using data from 35,028 participants and including 32,664,171 SNPs | Cardiovascular risk factors and diabetic factors are associated with a widespread atrophy pattern in the brains of the general population. Overexpression of genes associated with this widespread atrophy was discovered in the hippocampus and basal ganglia, as well as the heart. Genes associated with focal medial temporal lobe atrophy were not overexpressed in the heart. |
| (Zhang *et al.*, 2024) | Human brain transcriptome analysis and murine study | The *GNB5* and *SHC2* genes may be implicated in AD, which are associated with a developmental disorder with cardiac arrhythmia and sympathetic neuron loss, respectively. Expression of human *GNB5* in APPswe/PS1de9 mice increased Aβ and tau amyloidosis. |
| (Selvaraj *et al.*, 2022) | Human observational study in 15064 patients | The number of APOE-ε4 alleles did not increase the prevalence of heart failure or affect echocardiographic parameters. Aβ peptide levels negatively correlated with the number of APOE-ε4 alleles, and Aβ did not associate with the development of heart failure. |
| Autonomic dysregulation in Alzheimer’s disease | | |
| Alzheimer’s amyloidosis in brainstem autonomic nuclei | | |
| (Parvizi *et al.*, 2001) | Human observational study in 32 autopsy specimens | Aβ and tau amyloidosis affected the dorsal motor nucleus of the vagus, *nucleus tractus solitarius, nucleus ambiguus*, and the *locus coeruleus*, the main site of noradrenaline synthesis. The periaqueductal grey matter was affected only by Aβ plaques in most specimens. |
| (Tian *et al.*, 2022) | Human observational study in 9 autopsy specimens | Tauopathy found in the medulla oblongata in AD patients, especially the dorsal motor nucleus of the vagus, reticular nucleus, and *nucleus ambiguus*. Tauopathy predisposed to axonal swelling and axonopathy. |
| (Parvizi *et al.*, 2000) | Human observational study in 32 autopsy specimens | Aβ plaques and NFTs were present bilaterally in the periaqueductal grey in 81% of patients with AD. |
| (Rüb *et al.*, 2001) | Human observational study in 27 autopsy specimens | NFTs were stained in the medullary reticular formation and pontine nuclei of AD patients, including the medial parabrachial nucleus, lateral parabrachial nucleus, and subpeduncular nucleus. These nuclei demonstrated little Aβ staining. |
| (Guo *et al.*, 2016) | Human observational study in 11 autopsies | NFTs, but not Aβ plaques, were discovered in the spinal cords of patients with AD. |
| (Jacobs *et al.*, 2022) | Human observational study in 271 patients | Lower volumes of the midbrain, pons and medulla oblongata did not associate with cerebral tau deposition but did relate to increased Aβ deposition. The volume of the hippocampus did not relate to Aβ or tau deposition. |
| Sympathetic and parasympathetic nervous dysfunction in Alzheimer’s disease | | |
| (Lai *et al.*, 2019) | Murine study *in vivo* and *in vitro* study | A spinal cord electrophysiology study that showed that Aβ40 potentiated NMDA-induced depolarisations in sympathetic preganglionic neurons. The underlying mechanism involved phosphorylation of NMDA GluR1 subunits through protein kinase C pathway. |
| (Chen *et al.*, 2023a) | Murine study *in vivo* | In early-stage AD pathology, APPswe/PS1de9 mice exhibited reduced RR intervals and total power and high-frequency power of HRV during sleep. Mice with advanced AD pathology showed these changes during sleep and wakefulness. |
| (Karunungan *et al.*, 2023) | *In vitro* study | Inhibiting the PS1 and PS2 components of γ-secretase reduced dendrite formation and reduced dendrite length but did not affect axonal length. |
| (Tayler *et al.*, 2018) | Murine study *in vivo* | Aβ infusion increased blood pressure compared to saline infusion but did not affect heart rate. Aβ infusion reduced baroreflex gain but had no effect on HRV. |
| (Starmans *et al.*, 2024) | Human observational study in 518 patients | Diastolic blood pressure, nocturnal dipping (the ratio of nighttime blood pressure to daytime blood pressure), and orthostatic hypotension were not significantly associated with higher neurofilament light levels after multivariable analysis. These parameters were not significantly associated with Aβ or p-tau after multivariable analysis. |
| (Miller *et al.*, 2008) | Human observational study in 12 patients | p-tau amyloidosis was discovered in the cardiovascular reflex arc and baroreflex nuclei: medullary autonomic nuclei, the dorsal motor nucleus of the vagus, *nucleus tractus solitarius* and the *nucleus ambiguus*. Patients with carotid sinus hypersensitivity had greater cerebral tauopathy but no difference in Aβ amyloidosis compared to controls. |
| (Xue *et al.*, 2023) | Human observational study in 199 patients | Patients with obstructive sleep apnoea and mild cognitive impairment had lower HRV parameters, such as the mean of standard deviations of R-R intervals. AD biomarkers and complement activation partially mediated the influence of HRV on cognitive function. |
| (Díaz-Román *et al.*, 2021) | Human observational study in 57 patients | In patients with mild cognitive impairment, a greater apnoea/hypopnoea index, indicating more severe obstructive sleep apnoea, was shown to predispose to greater tau biomarkers in the CSF. |
| (Lohman *et al.*, 2024) | Human observational study in 122 patients | In patients without dementia or other neurological disorders, reduced connectivity within the central autonomic network, as well as its parasympathetic and sympathetic arms, correlated to reduced Aβ42/Aβ40 ratios. Likewise, connectivity in these networks inversely correlated with plasma GFAP. Reduced connectivity in the general central autonomic network, but not the individual parasympathetic or sympathetic networks, negatively correlated with plasma neurofilament light, but after adjusting for age, sex and ApoE-ε4 this became non-significant. |
| (Molloy *et al.*, 2023) | Human observational study in 179 patients | In patients with abnormal amyloid/tau rations, a higher resting heart rate correlated to worse performance on MMSE. This was not present for patients with normal amyloid/tau ratios, mild cognitive impairment or AD. Greater HRV was associated with better executive function in patients with normal amyloid/tau ratio, but no such association was present in patients with abnormal amyloid/tau ratios. |
| (Arechavala *et al.*, 2021) | Human observational study in 46 patients | Participants with abnormal amyloid/tau ratios showed a reduced low frequency heart rate variability during task switching. This was not observed in participants with normal amyloid/tau ratios. There was a trend for a reduction in the standard deviation of R-R intervals in patients with abnormal amyloid/tau ratios. |
| (Santos *et al.*, 2017) | Human observational study in 63 patients | Patients with no cerebral Aβ deposition showed an increase in vagal ratio and respiratory sinus arrhythmia during task induced cognitive stress whereas patients with cerebral Aβ deposition did not show these changes. |
| (Min *et al.*, 2023) | Randomised clinical trial in 108 patients | Using daily breathing techniques to increase HRV reduced Aβ42 and Aβ40 compared to pre-intervention. Using different breathing techniques to decrease HRV increased Aβ peptides and reduced p-tau/t-tau ratio, which were associated with CREB downregulation, implying down-regulation of β-adrenergic signalling. |
| Hormonal alterations in Alzheimer’s disease | | |
| HPA axis dysfunction relates to cardiovascular sequelae of Alzheimer’s disease | | |
| (Baloyannis *et al.*, 2015) | Human observational study in 24 patients | The suprachiasmatic, supraoptic and paraventricular nuclei in patients with AD demonstrated reduced neuronal populations, reduced dendrite formation, smaller mitochondria, and more atrophic Golgi apparatus versus controls. |
| (Wang *et al.*, 2016) | Murine study *in vivo* | Aβ31-35 disrupted the circadian rhythm of mice, causing them to have abnormal moving and resting phases during a wheel-running activity in constant darkness, compared to control mice. Aβ31-35 disrupted circadian oscillations of Per1 and Per2 in the suprachiasmatic nucleus, hippocampus and the heart, compared to control mice. |
| (Hebda-Bauer *et al.*, 2013) | Murine study *in vivo* and *in vitro* study | Male triple transgenic mice expressing the PS1M146V, APPswe and tauP301L transgenes demonstrated increased glucocorticoid receptors and reduced corticotrophin-releasing hormone in the paraventricular nucleus compared to wild-type mice. Plasma corticosterone levels were similar in all mice. |
| (Pedersen *et al.*, 1999) | Murine study *in vivo* | Mice expressing the Swedish mutation APP were unable to maintain normal blood glucose concentrations under stress and demonstrated increased corticosterone levels. AD mice did not show poor insulin function compared to wild-type mice. |
| (Morgese *et al.*, 2014) | Murine study *in vivo* | Aβ administration led to poorer performance on the passive avoidance test, implying memory impairment, increased cerebral noradrenaline, and reduced circulating corticosterone compared to rats given a sham injection. |
| (Touma *et al.*, 2004) | Murine study *in vivo* | Faecal corticosterone metabolites and plasma corticosterone were higher in mice expressing APP mutations compared to wild-type mice. This occurred 45 days after the start of the experiment in males and after 90 days in females. No differences in the sympathetic-adrenomedullary system were observed. Aβ plaque burden did not correlate to hormone levels. |
| (Lv *et al.*, 2020) | Murine study *in vivo* | Aβ42 administration reduced performance on the Morris water maze and passive avoidance tests and increased adrenal gland weight, corticosterone levels, glucocorticoid receptor expression, and corticotrophin-releasing hormone receptor expression. |
| (Hendrickx *et al.*, 2021) | Murine study *in vivo* | Mice overexpressing APP demonstrated significantly increased blood corticosterone, which correlated to increased pulse wave velocity, increased blood pressure and urinary adrenaline/noradrenaline metabolites versus wild-type mice. APP overexpression reduced α1-adrenoceptor-mediated aortic contraction. These effects were independent of local Aβ amyloidosis, and no alteration in echocardiographic parameters was found. |
| (Popp *et al.*, 2015) | Human observational study in 147 patients | CSF cortisol concentrations were higher in patients with AD compared to cognitively normal subjects and predisposed to more rapid AD progression and cognitive decline independent of Aβ42 and t-tau. |
| (Pietrzak *et al.*, 2017) | Human observational study in 416 patients | Higher cortisol levels associated with lower episodic memory and executive function. Aβ-positive adults with normal cognition and hypercortisolaemia demonstrated greater rates of cognitive decline than adults with low cortisol levels. |
| (Vasantharekha *et al.*, 2024) | Human observational study in 3126 patients | Patients with AD demonstrated higher serum APP and cortisol levels than controls and those with mild cognitive impairment, which predisposed to lower MMSE scores. Phosphorylated protein kinase B and extracellular signal-regulated kinase were reduced in patients with AD initially, but increased during follow-up, perhaps providing a mechanism for cortisol-mediated cognitive impairment. |
| Alzheimer’s pathology downregulates the effects of melatonin | | |
| (Cecon *et al.*, 2015) | *In vitro* study | Aβ40 or Aβ42 reduced pineal gland melatonin synthesis, mediated by NF-КB pathway upregulation. Aβ42, but not Aβ40, reduced the number of melatonin receptors on endothelial cells and their ligand binding sites. Aβ peptides interrupted ERK1/2 signalling utilised by melatonin receptors. |
| (Wang *et al.*, 2020) | Human observational study and murine study *in vivo* | In AD patients, the rate of pressure increase during ventricular contraction was lower alongside reduced melatonin levels and left ventricular diastolic pressure was increased. APP/PS1 mice exhibited reduced melatonin levels, reduced fractional shortening, atrophic remodelling, impaired cardiomyocyte contraction and intracellular Ca^2+^ abnormalities. Melatonin supplementation reversed these effects. AD patients and APPswe/PS1de9 mice show reduced mitochondrial aldehyde dehydrogenase activity. |
| (Feng *et al.*, 2004) | Murine study *in vivo* | Supplementation of 10mg/kg/day of melatonin for 4 months alleviated deficit in learning and memory in APP transgenic mice, through reducing cerebral Aβ amyloidosis as well as having antioxidant and anti-apoptotic effects. Furthermore, melatonin increased choline acetyltransferase activity in the frontal cortex and hippocampus, showing the protective effects of melatonin on cholinergic pathways in the brain. |
